# Flexible Ni/NiO_x_-Based Sensor for Human Breath Detection

**DOI:** 10.3390/ma15010047

**Published:** 2021-12-22

**Authors:** Le Duc-Anh Ho, Vu Binh Nam, Daeho Lee

**Affiliations:** Laser and Thermal Engineering Laboratory, Department of Mechanical Engineering, Gachon University, Seongnam 13120, Korea; holeducanh31895@gmail.com (L.D.-A.H.); vubinhnam@gmail.com (V.B.N.)

**Keywords:** NiO_x_, nickel oxide, Ni, nickel, nanoparticles, laser digital patterning, laser-induced reductive sintering process, breath sensor, flexible sensor

## Abstract

We developed a simple methodology to fabricate an Ni/NiO_x_-based flexible breath sensor by a single-step laser digital patterning process of solution-processed NiO_x_ thin-film deposited using NiO_x_ nanoparticle ink. Laser-induced reductive sintering phenomenon enables for the generation of three parts of Ni electrodes and two narrow NiO_x_-sensing channels in between, defined on a single layer on a thin flexible polymer substrate. The Ni/NiO_x_-based breath sensor efficiently detects human breath at a relatively low operating temperature (50 °C) with fast response/recovery times (1.4 s/1.7 s) and excellent repeatability. The mechanism of the gas-sensing ability enhancement of the sensor was investigated by X-ray photoelectron spectroscopy analysis. Furthermore, by decoupling of the temperature effect from the breathing gas, the response of the sensor due to the temperature alone and due to the chemical components in the breathing gas could be separately evaluated. Finally, bending and cyclic bending tests (10,000 cycles) demonstrated the superior mechanical stability of the flexible breath sensor.

## 1. Introduction

Human breath, one of the most important physiological characteristics of the human body, can provide critical information for the symptoms of pulmonary [1] and cardiac issues [2]. Thus, breath monitoring is considered a feasible method for human health checks. Non-invasive breath analysis is an efficient and economical method for the detection of diseases and is considered an alternative approach to blood analysis for potential applications in metabolic control, disease diagnosis, and respiratory status monitoring [3,4,5,6]. In recent years, numerous breath sensors based on humidity sensors [7,8,9], gas sensors [10,11,12,13], and optical sensors [14,15] have been created using various methods such as chemical acid etching, conventional evaporation, and spray pyrolysis reactors. However, these breath sensors have limitations in their actual utilization as flexible breath sensors, because they require a high manufacturing temperature, and the etching chemicals used in the fabrication processes may damage flexible polymer substrates. Motivated by the rapid development of wearable devices [16,17,18,19,20] and sensors with advances in flexibility [20,21,22,23], non-invasiveness and real-time monitoring have drawn significant attention. To meet these requirements, a novel method is required to enable the direct deposition and patterning of sensors onto flexible polymer substrates.

The laser direct patterning (LDP) process, which enables electrode patterning without the photolithography process, has been extensively developed to fabricate flexible/stretchable devices due to the fact of its simple yet high-precision process and low thermal stress applied to heat-sensitive substrates [16,24,25,26,27,28,29,30]. Nanoparticle inks, including various nanomaterials, such as Au [31,32], Ag [33,34], CuO_x_ [35,36], Cu [37,38,39], and NiO_x_ [40,41], have been widely utilized in the process to produce conductor or semiconductor electrodes. In our previous studies, flexible Ni and/or NiO_x_ electrodes fabricated by the LDP process, which induces reductive sintering of the NiO_x_ thin film owing to the reaction between nanoparticles and the reducing agents in the nanoparticle ink, have been applied to various flexible devices including temperature sensors, touchscreen panels, and high-temperature heaters [16,24,26,42,43,44]. Recently, NiO_x_ nanostructures have drawn significant attention for gas-sensing applications owing to their high specific surface areas, excellent reproducibility, high sensitivity, and low price [45,46,47]. However, to date, studies on the LDP process of NiO_x_ to fabricate a flexible breath sensor have not yet been reported.

In this study, we developed a simple LDP process for a solution-processed NiO_x_ NP thin film to fabricate a flexible Ni/NiO_x_-based breath sensor on a thin flexible polyimide (PI) substrate (thickness ~50 µm). The sensor comprises three parts of Ni electrodes and two narrow NiO_x_ sensing channels in between, on a single layer. The effect of the laser-induced reductive sintering (LRS) phenomenon on the sensor performance was evaluated using X-ray photoelectron spectroscopy (XPS) analysis. The flexible Ni/NiO_x_-based breath sensor exhibited fast response and recovery times to human breath even at a low operating temperature. Furthermore, the responses of the breath sensor to various stimuli, such as temperature, CO_2_, and humidity, were also investigated to identify the reactivity of each component in the breathing gas. Finally, the superior mechanical stability of the sensor on the PI substrate was confirmed through bending and cyclic bending tests (up to 10,000 cycles).

## 2. Materials and Methods

### 2.1. NiO_x_ NP Synthesis and NiOx Thin-Film Deposition

NiO_x_ NP ink and its thin film were prepared following a previous study with a few modifications [16]. Nickel(II) nitrate hexahydrate (Ni(NO_3_)_2_∙6H_2_O), sodium hydroxide (NaOH), and cetyltrimethylammonium bromide (CTAB, C_19_H_42_BrN) were supplied by Alfa Aesar, Haverhill, MA, USA. 1-Pentanol (C_5_H_12_O) and polyvinylpyrrolidone (PVP, (C_6_H_9_NO)_n_, molecular weight: 10,000) were provided by Sigma–Aldrich, St. Louis, MO, USA. All chemicals were used without further purification. Ni(NO_3_)_2_∙6H_2_O of 0.1 mol was dissolved in 100 mL of deionized (DI) water, and the pH of the solution was adjusted to 10 by adding NaOH solution (10 M) dropwise to produce Ni(OH)_2_. The colloidal Ni(OH)_2_ of light green color was washed with DI water several times, centrifuged at 3000 rpm for 5 min, and then dried at 80 °C for 6 h. The dried Ni(OH)_2_ was thermally decomposed to dark-black nonstoichiometric NiO_x_ NPs by calcination at 270 °C for 2 h in a convection oven by the following reaction [48]:

Ni(OH)_2_ → NiO_x_ + H_2_O (1)

The NiO_x_ NPs (31 wt%) dispersed in 1-pentanol (61.7 wt%) with PVP (6.8 wt%) and CTAB (0.5 wt%). After 10 h ultrasonication (Powersonic 610, Hwashin, Gwangju-si, Korea), well-dispersed NiO_x_ NP ink was prepared. The PI substrate was supplied from Isoflex KESPI, Gyeonggi-do, Korea. A uniform NiO_x_ thin film on a PI substrate was prepared by spin coating at 1000 rpm for 60 s as shown in Figure S1, Supplementary Materials.

### 2.2. Laser Digital Patterning Process

The laser setup for the LDP process was applied to the as-synthesized NiO_x_ thin film using a 532 nm continuous-wave Nd:YVO_4_ laser (Sprout-G, Lighthouse photonics, San Jose, CA, USA) as illustrated in Figure S2, Supplementary Materials. The spot diameter of the focused laser beam was approximately 20 µm and scanned on the thin film using the galvanometer scanner (HurrySCAN III, Scanlab, Puchheim, Germany). To find the optimal laser power to generate the sensor electrodes, the laser power was precisely controlled by rotating the half-wave plate installed in front of the polarized beam splitter.

### 2.3. Characterizations

The size and crystal structure of the synthesized NiO_x_ NPs were measured using transmission electron microscopy (TEM, JEOL JEM-2100F, Tokyo, Japan). The surface morphology was characterized using scanning electron microscopy (SEM, Hitachi S-4800, Tokyo, Japan). Energy-dispersive X-ray spectrometry (EDS, Hitachi S-4800, Tokyo, Japan) was conducted to investigate the elemental chemical compositions. The electrical resistance was measured using a Digital Source Meter (Keithley 2450, Cleveland, OH, USA), and the operating temperature was established using a hot plate (Fisher Scientific, Hampton, NH, USA). The temperature of the hot plate was precisely controlled using a commercial thermocouple (type K, EA11A, Extech Instruments, Nashua, NH, USA). X-ray photoelectron spectroscopy (XPS) measurements were conducted using an FC-XP10 (Nexsa, Thermo Fisher Scientific, Waltham, MA, USA). The thicknesses of the thin films were characterized using atomic force microscopy (AFM, Park System XE100, Suwon, Korea).

## 3. Results and Discussion

**Figure 1:**
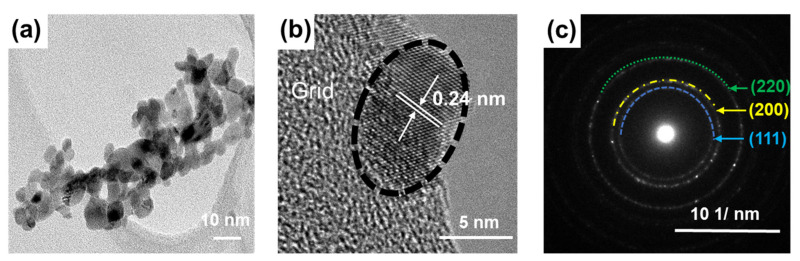
Transmittance electron microscopy (TEM) image (**a**), high-resolution TEM (HR-TEM) image (**b**), and selective-area electron diffraction (SAED) pattern (**c**) of the NiO_x_ nanoparticles.

The size of the as-synthesized NiO_x_ NPs determined by TEM (Figure 1a) was in the range of 4–10 nm. The high-resolution TEM image (Figure 1b) and the selected area electron diffraction (SAED) pattern (Figure 1c) showed that NiO_x_ NPs have a cubic crystalline structure and the spacing between two adjacent fringes was 0.24 nm, which corresponded to the (111) plane of the NiO_x_.

**Figure 2:**
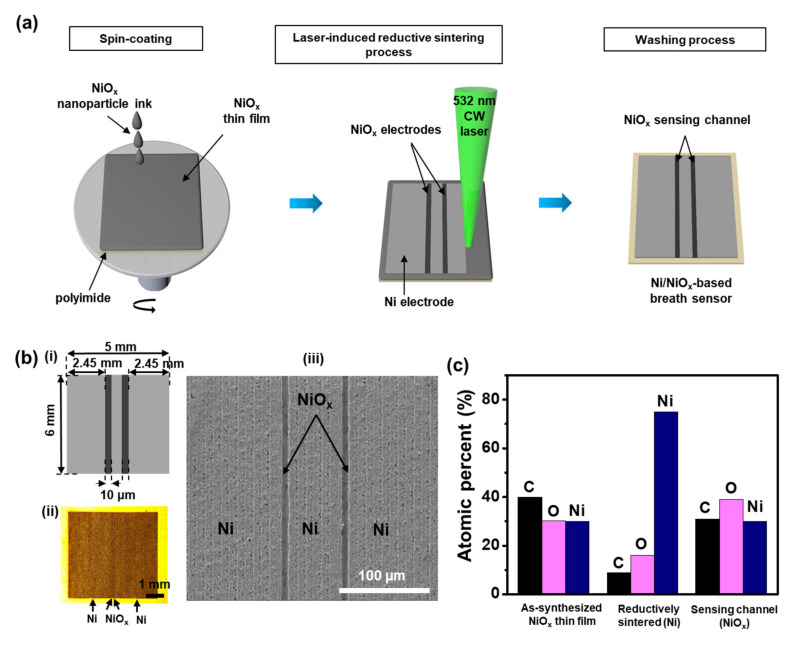
(**a**) Schematic of the fabrication procedures of the flexible Ni/NiO_x_-based breath sensor on polyimide (PI). (**b**) (i) Schematic drawing, (ii) optical microscopy image, and (iii) scanning electron microscopy (SEM) image of the Ni/NiO_x_-based breath sensor. (**c**) Chemical compositions of the as-synthesized NiO_x_ thin film, reductive sintering Ni electrode, and the NiO_x_-sensing channel, analyzed by energy-dispersive X-ray spectrometry.

Figure 2a illustrates the entire process step to fabricate the flexible Ni/NiO_x_-based breath sensor. The sensor consists of three Ni electrodes and two narrow NiO_x_-sensing channels in between, which was constructed by the LRS phenomenon during the LDP process on the NiO_x_ NP thin film. First, an NiO_x_ NP thin film was coated on a PI substrate by spin coating. After drying under ambient conditions, the laser irradiation was applied to the NiO_x_ thin film to produce Ni electrodes at the power density of 6.4 kW cm^−2^ with a scanning speed of 50 mm s^−1^; details of the mechanism by which Ni electrodes were generated by the LRS phenomenon of NiO_x_ upon laser irradiation are described in the previous research results [43]. Based on the observation that a single stroke of laser scan generates a 22 μm wide Ni electrode (Figure S3, Supplementary Materials), a 15 μm scan pitch was selected to allow an area of each Ni electrode to overlap, thereby generating continuous Ni electrode areas. The NiO_x_ sensing channels could be generated without direct laser irradiation; after the last laser scan of the left side of the Ni electrode area is finished, multiple scans were performed in the laser-off state to move the laser beam position without applying laser irradiation; then, laser scanning was continued again in the laser-on state to create the center Ni electrode. In this process, the annealed and consolidated NiO_x_ area was instantaneously generated between the two nickel electrodes by thermal diffusion. The same procedure was applied between the last laser scan for the center Ni electrode and the first laser scan of the right-side Ni electrode. Note that two NiO_x_ channels were fabricated to ensure the liable performance of the sensor in this study, and the number of NiO_x_-sensing channels was easily controllable by repeating the procedure as mentioned above. Finally, DI water was used to wash the unirradiated and unconsolidated parts while the laser-irradiated Ni parts and their adjacent NiO_x_ parts were strongly adhered to the PI substrate, which completed the device fabrication process. It is also worth noting that the entire process for the sensor fabrication was conducted under ambient conditions, and the Ni electrodes for current collectors and the NiO_x_-sensing channels were created as a single layer, greatly simplifying the fabrication process. The size of the sensor was 6 × 5 mm^2^, while the size of each sensing channel was measured at approximately 10 μm. Figure 2b(i,ii) show the schematic and microscopic image of the fabricated sensor, respectively, and Figure 2b(iii) displays an SEM image of the sensor near the sensing channel; the high magnification SEM images for the as-synthesized NiO_x_ thin film, the NiO_x_-sensing channel, and the Ni electrode are displayed in Figures S4 and S5, Supplementary Materials. The thickness of Ni electrode parts was determined to be approximately 600 nm, whereas the thickness of annealed NiO_x_ parts was measured to be approximately 330 nm as verified by the AFM image of the single Ni/NiO_x_ electrode (Figure S3, Supplementary Materials). To identify the change in the chemical element compositions by laser processing, EDS analysis was performed. The atomic ratio C, O, and Ni was 40:30.1:29.9 for the as-synthesized thin film, whereas the values of the Ni electrode and the NiO_x_-sensing channel were 9:16:75 and 31:39:30, respectively (Figure 2c). The increase in the Ni peak and the decrease in the C peak in the Ni electrode confirmed that the NiO_x_ converted to Ni by thermal decomposition of PVP by the LRS phenomenon.

**Figure 3:**
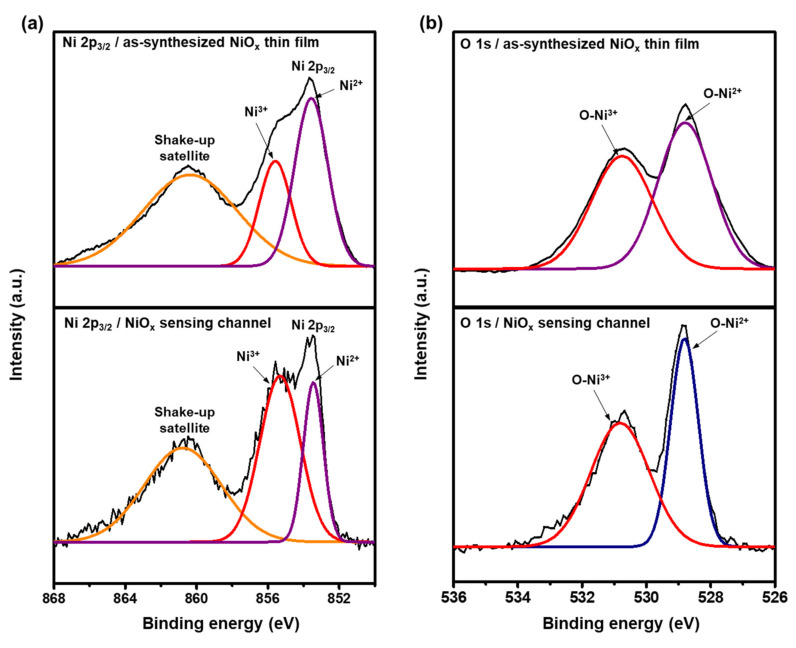
X-ray photoelectron spectroscopy (XPS) results from the as-synthesized NiO_x_ thin film and the NiO_x_-sensing channel of the breath sensor for (**a**) Ni 2p_3/2_ and (**b**) O 1s.

XPS analyses were conducted to compare the compositions and chemical states of the as-synthesized NiO_x_ thin film with the NiO_x_-sensing channel generated during the LDP process. It is worth noting that the 10 µm wide sensing channel could not be directly used for XPS analyses, because the minimum X-ray beam size employed for the XPS measurements was 25 µm. Therefore, for the XPS measurements, the sensor with the wider sensing channel (~50 µm) was fabricated by producing the thicker thin film and subsequent LDP process. Figure 3 shows the XPS profiles and deconvoluted Gaussian peaks of Ni 2p_3/2_ and O 1s for the as-synthesized thin film and the sensing channel. The Ni 2p_3/2_ peaks at 853.6 eV and the O 1s peaks at 528.8 eV were assigned to Ni^2+^ and were attributed to the Ni–O octahedral binding of the cubic rock–salt NiO structure [49,50,51]. The Ni 2p_3/2_ peaks at 855.6 eV and the O 1s peaks at 530.8 eV were associated with the Ni^3+^ [50,52]. The satellite Ni 2p_3/2_ peaks at 860.8 eV were observed because of the shake-up process in the NiO_x_ structure [52]. The XPS analyses clearly show that there was a significant increase in the Ni^3+^ states in the NiO_x_-sensing channel compared to that of the as-synthesized NiO_x_ thin film. This could be attributed to the rapid thermal annealing of the NiO_x_-sensing channel by heat diffusion from the region directly irradiated by laser. Absorbed laser increases the temperature of the material to very high temperature in an extremely short time, and thermal diffusion happens due to the temperature gradient. The temperature induced in the NiO_x_-sensing channel by thermal diffusion without direct laser irradiation is not sufficient to reductively sinter NiO_x_ to Ni. However, NiO_x_ in the sensing channel was still subjected to rapid thermal annealing in a reducing atmosphere due to the decomposition of PVP that existed in the NiO_x_ layer, i.e., in a situation where oxygen is insufficient, resulting in an increase in oxygen defects in NiO_x_. As a result, it is postulated that Ni^3+^ increased to maintain charge neutrality. We believe that the Ni^3+^ increase in the NiO_x_-sensing channel can contribute to the enhancement of the breath-sensing ability due to the following reasons: The increase in the Ni^3+^ states led to an increase in hole concentration in the hall accumulation layer on the NiO_x_ surface. As the sensor is exposed to human breath, the absorbed water molecules from the breathing gas under the applied electricity are ionized into H_3_O^+^ and free electrons [53,54,55]. These electrons are injected into the NiO_x_, and the resultant hole concentration in the hole accumulation layer on the surface of the NiO_x_ decrease via electron–hole recombination, thereby increasing the sensor’s resistance [56]. It is noteworthy that NiO_x_ is known as a p-type semiconductor. On the other hand, H_3_O^+^ could act as the charge carriers by proton hopping [55,57], which leads to a decrease in the resistance. Therefore, the electron injection mechanism and proton hopping mechanism have opposite effects on resistance change. Considering that the experimental results showed a resistance increase when humidity was applied, it can be postulated that hole concentration decreased in the hole accumulation layer of the NiO_x_ due to the fact that electron injection was the more dominant mechanism in the humidity sensing of the sensor (Figure S6, Supplementary Materials).

**Figure 4:**
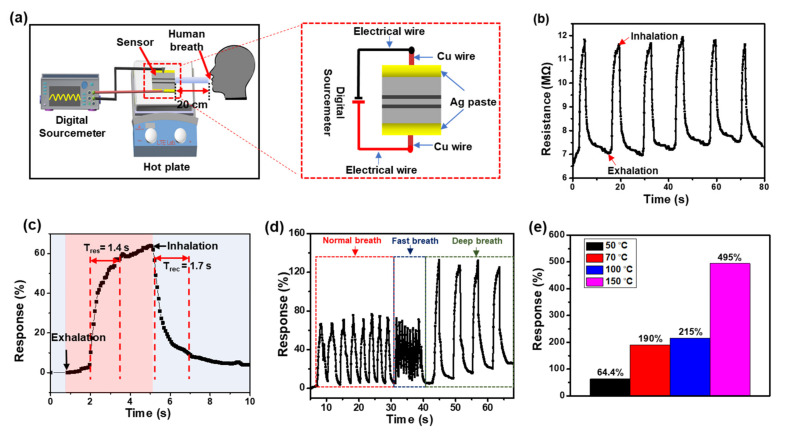
(**a**) Schematic diagram of the experimental setup to test the breath sensing performance of the senor. (**b**) Electrical resistance variation of the Ni/NiO_x_-based breath sensor to the normal breathing rate at the operating temperature of 50 °C. (**c**) Response and recovery times of the breath sensor to the normal breath rate at 50 °C. (**d**) Response curves of the breath sensor to the different breathing rates at 50 °C. (**e**) Maximum responses of the breath sensor to the normal breath rate at different temperatures.

The experimental setup for the breath-sensing performance is illustrated in Figure 4a. The sensor was placed on a hot plate to set the sensor’s operating temperature, while the human breath was blown to the sensor through a straw at a distance of 20 cm. The real-time electrical resistance variation of the sensor to the human breath was recorded using a digital source meter. Conductive silver paste was brushed onto both ends of the Ni/NiO_x_ electrodes, and Cu wires were used for the connection between the sensor and the source meter. From room temperature (23 °C) to 40 °C, the resistance changes of the sensor to the breath did not show specific tendency. This was probably because the effects of temperature changes and gas molecules on resistance changes due to the breathing tended to be comparable in opposites way and, thus, compensated each other in this temperature range. Figure 4b shows the electrical resistance variations of the sensor corresponding to the six-time breathing cycles over a period of 80 s at the operating temperature of 50 °C. The footage for this test is provided in Video S1, Supplementary Materials. During the test, the resistance changes for each cycle was not the same, because the depth and amount of human breathing was not always constant. The response of the sensor was calculated using the following equation [58]:

(2) $$\mathrm{Response}\;(\%)=\frac{R-R_{o}}{R_{o}}\;\times \;100$$

where *R*_o_ and *R* represent the electrical resistances of the sensor before and after applying the human breath, respectively. The electrical resistance of the sensor increased from 7.3 MΩ to 12 MΩ during the first exhalation of the breath and returned to its original value during inhalation. The maximum response of the Ni/NiO_x_-based breath sensor at the operating temperature of 50 °C was calculated to be approximately 64%. The response and the recovery times of the sensor, described as the time taken to change the signal between the designated lower and upper limits of 10% and 90%, respectively, were determined to be 1.4 s and 1.7 s to the normal breathing, respectively, as shown in Figure 4c. The response and the recovery times of our breath sensor were faster than those of other breath sensors published in the previous literature [59,60]. The fast response of the sensor can be attributed to the thin thickness of the Ni/NiO_x_ electrodes and the low heat capacity of the thin PI substrate [26,43]. The performance comparison of the breath sensors for different materials reported in the previous literature and in this study is presented in Table S1 and Figure S7 in the Supplementary Materials. The breath sensor could also detect a faster breathing rate and a deep breath as shown in Figure 4d. The changes in the sensor signals for various breathing rates mean that sensors can monitor the regularity or irregularity of breathing. Figure 4e displays the maximum response values of the sensor as a function of operating temperature. The response values increase with increasing operating temperature because more water molecules are adsorbed on the surface of NiO_x_ at a higher temperature.

**Figure 5:**
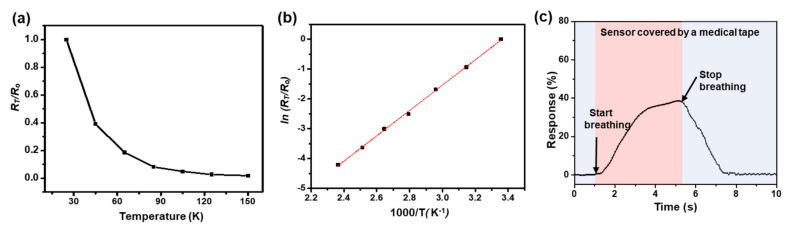
(**a**) Electrical resistance variation of the breath sensor corresponding to temperature change only. (**b**) Data fitting to determine the thermal sensitivity index (B-value) of the breath sensor. (**c**) Response curve of the breath sensor covered by a medical tape to the normal breath rate at 50 °C.

The breath-sensing capability of the Ni/NiO_x_-based sensor was attributed to a combined response of the sensor to temperature change by breathing and chemical components in the breath. To distinguish the reaction of the sensor to temperature change only, a 20 µm thick medical tape (Tegaderm, 3M, Saint Paul, MN, USA) was attached onto the surface of the sensor for encapsulation, which effectively blocked penetration of chemical molecules from the breathing gas to the sensor, while the thermal effect could still reach the sensor’s surface. Then, the electrical resistance variation of the breath sensor at different temperatures (from 23 to 150 °C) was recorded by placing the sensor on a hot plate. For a precise measurement, the surface temperature of the hot plate was monitored using a calibrated commercial thermocouple probe (type K, EA11A). The electrical resistance of the Ni/NiO_x_-based breath sensor decreased as the temperature increased (Figure 5a), showing a typical characteristic of a negative temperature coefficient (NTC) thermistor, which can be described by the following Arrhenius relation [61]:

(3) $$R_{T}=R_{o}e^{B\left(\frac{1}{T}-\frac{1}{T_{o}}\right)}$$

where *R*_T_ and *R*_o_ are the electrical resistance at temperature *T* and *T*_o_, respectively, and *B* is the thermal sensitivity index expressed in K. Equation (3) can also be rewritten as ln($\frac{R_{T}}{R_{o}}$) = *B*($\frac{1}{T}-\frac{1}{T_{o}}$), where the linear relationship between ln($\frac{R_{T}}{R_{o}}$*)* and 1/*T* is established. Figure 5b shows the same data as in Figure 5a plotted on different axis scales. By data fitting, the *B*-value was calculated to be approximately 4190 K for the overall measurement range (25–150 °C). The maximum response of the breath sensor covered by the medical tape was 38.0% at 50 °C (Figure 5c). Considering that the temperature of normal exhaled breath is approximately 34 °C, it should be noted that the sensor was subjected to cooling and, thus, the resistance of the sensor increased, which result in a positive response value. We also investigate trends in resistance change of the sensor when CO_2_ gas and humidity were applied on the non-encapsulated sensor, because they are the main gas components that differentiate exhaled gas from atmospheric air. As provided in Figure S7, Supplementary Materials, the sensor resistance increased for both cases. Even though we did not control the exact amount of CO_2_ gas and humidity that corresponded to actual breathing, this study clearly showed that CO_2_ and humidity can contribute to the resistance increase of the breath sensor upon exhalation. Therefore, considering the response values of the sensor without and with encapsulation (64% and 38%) of the sensor at the operating temperature of 50 °C, the response of the breath sensor to stimuli other than temperature during breathing is expected to be 26%.

**Figure 6:**
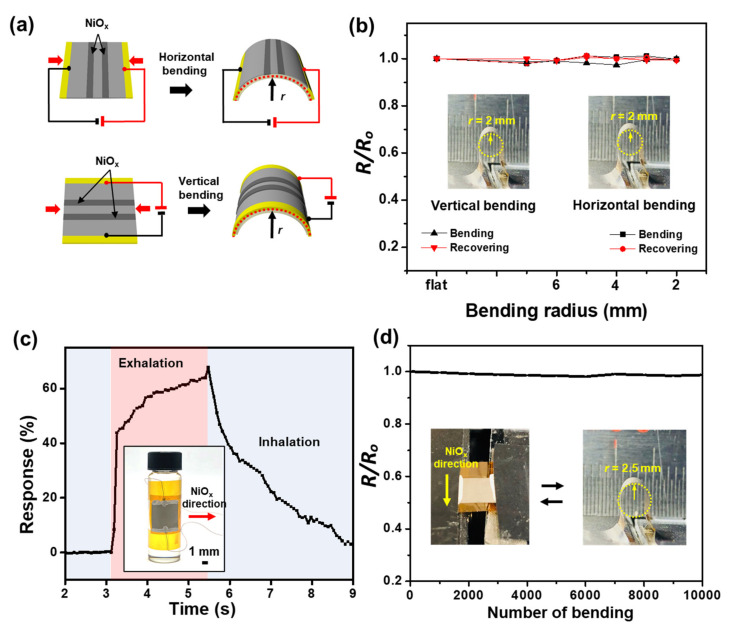
(**a**) Schematic illustration of the horizontal bending and vertical bending states of the sensor. (**b**) Electrical resistance change (*R/R*_o_) of the breath sensor under various bending radii under each bending state. (**c**) Response curve of the Ni/NiO_x_-based breath sensor to the normal breathing rate at the operating temperature of 50 °C under a bending condition. (**d**) Relative resistance changes of the breath sensor during a cyclic bending test (10,000 cycles).

High electromechanical stability of the breath sensor is required as a flexible sensor that can be utilized under various circumstances. Figure 6a illustrates the schematic situations of horizontal bending (top) and vertical bending (bottom) of the sensor which are defined as the relative direction of the sensing channel to the bending. To perform a bending test, one side of the sensor was fixed at a position, while the other side was connected to a motorized linear stage to reach the designated bending conditions. The electrical resistance change (*R/R*_o_) of the breath sensor under various bending radii was examined, where *R*_o_ and *R* are the initial resistance and the resistance under bending conditions, respectively. Under both horizontal and vertical bending without any surface passivation, the resultant resistance changes were less than 3% up to a bending radius of 2 mm as shown in Figure 6b. This demonstrates the strong electromechanical stability of both Ni electrodes and the NiO_x_-sensing channels owing to strong adhesion and lowered bending stress of the electrodes on the thin PI substrate and the lowered bending stress. The performance of the Ni/NiO_x_-based breath sensor under a bending state was evaluated by attaching the sensor on the curved surface of a glass vial (diameter: 10 mm). The uniform temperature distribution of the sensor at the operating temperature on the curved surface could be achieved by filling the glass vial with silicon oil (3 mL) and heating on a hot plate. The maximum response (~68%) of the sensor to human breath under the bending condition was similar to that of the sensor in a flat state (Figure 6c). To examine the long-term stability of the sensor, a cyclic bending test was performed by moving the motorized stage back and forth repeatedly at a velocity of 20 mm s^−1^ to generate the horizontal bending conditions of the sensor up to a radius curvature of 2.5 mm, while the electrical resistance was measured at each flat state after release (Figure 6d). The resultant electrical resistance variation (*R*/*R*_o_) after 10,000 bending cycles was less than 3% confirming the long-term flexibility of the breath sensor.

## 4. Conclusions

A flexible Ni/NiO_x_-based breath sensor consisting of three parts of Ni electrodes and two narrow NiO_x_-sensing channels on a single layer that was generated by the LRS phenomenon during the LDP process of the solution-processed NiO_x_ thin-film deposited using the NiO_x_ NP ink. The sensor had excellent sensitivity with fast response/recovery times (1.4 s/1.7 s) at a relatively low operating temperature (50 °C) and shows strong electromechanical stability under bending conditions and during the cyclic bending test. By decoupling the temperature effect from the breathing gas, the response of the sensor, due to the temperature alone and due to the chemical components in the breathing gas, could be separately evaluated. The entire sensor fabrication process was conducted in an atmospheric environment without any vacuum equipment. Furthermore, no photolithographic process or alignment procedures were required to define the current collectors and the sensing channels separately. Therefore, we believe that this novel, yet simple laser process provides a new method of fabrication of flexible breath sensors by replacing existing fabrication methods and materials.

## Supplementary Materials

The following are available online at https://www.mdpi.com/article/10.3390/ma15010047/s1: Figure S1: A uniform NiO_x_ NP thin-film spin coat on a polyimide substrate; Figure S2: Schematic illustration of the experimental setup for the laser direct patterning process; Figure S3: Atomic force microscopy (AFM) image of the single Ni/NiO_x_ electrode; Figure S4: SEM images of (a) the as-synthesized NiO_x_ thin film and (b) NiO_x_ as the sensing channel; Figure S5: SEM image of the sensor at a high magnification; Figure S6: Schematic illustration of humidity sensing mechanism; Figure S7a: Response of the breath sensor to CO_2_ gas: Figure S7b: Response of the breath sensor to humidity; Table S1: Performance comparison of the breath sensors for different materials reported in the literature and in this study: Video S1: A video clip showing the response of the sensor to the human breath.
