# Supplementary material for: Flexible Ni/NiOx-Based Sensor for Human Breath Detection
Source: Materials (Basel). 2021 Dec 22;15(1):47. doi: 10.3390/ma15010047 (PMC8746032; doi:10.3390/ma15010047)
Supplement: Supplementary file 1 [file materials-15-00047-s001.zip › Supporting Information revised.pdf]

Supporting Information:

Flexible Ni/NiO_x-based Sensor for Human Breath Detection

Le Duc-Anh Ho†, Vu Binh Nam†, Daeho Lee*

[1]Laser and Thermal Engineering Laboratory, Department of Mechanical Engineering, Gachon University, Seongnam 13120, Korea.

* Correspondence: dhl@gachon.ac.kr

† L.D-A.H and V.B.N. These authors contributed equally to this work.

1. Uniform NiO_x thin film on a polyimide substrate

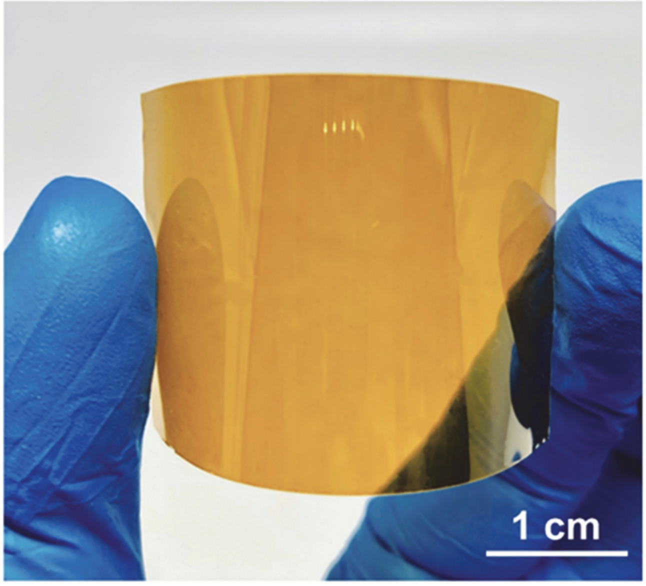

Figure S1. A uniform NiO_x NP thin film spin coated on a polyimide substrate

2. Schematic of the laser setup for the laser direct patterning process.

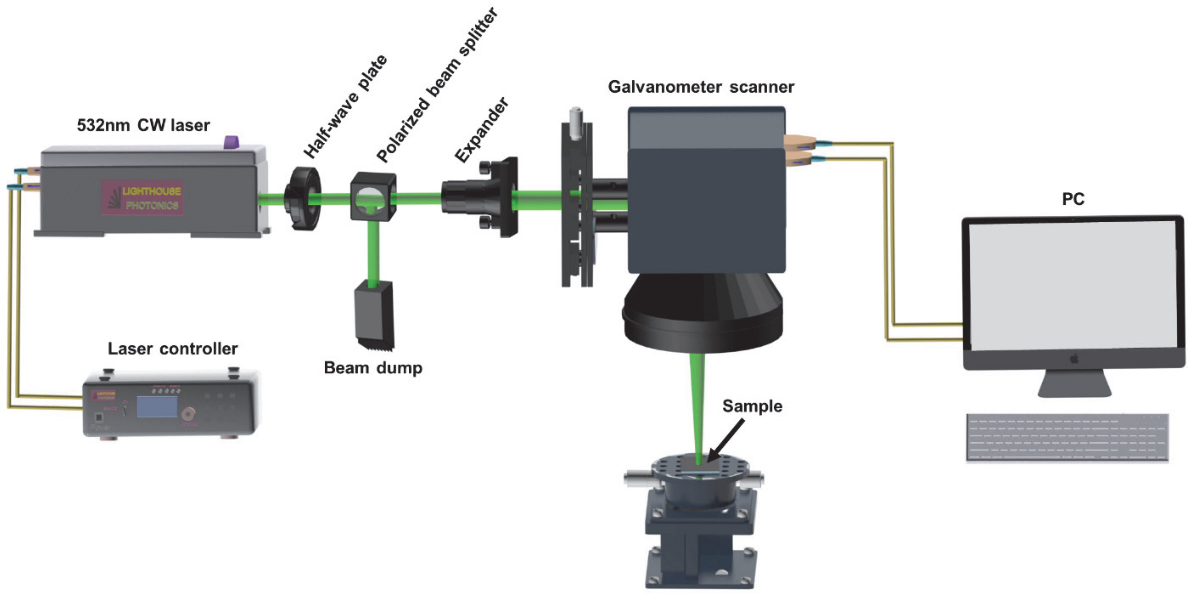

Figure S2. Schematic illustration of the experimental setup for the laser direct patterning process

3. Atomic force microscopy (AFM) image of the Ni/NiO$_x$ electrode generated by laser-induced reductive sintering process during the laser digital patterning process

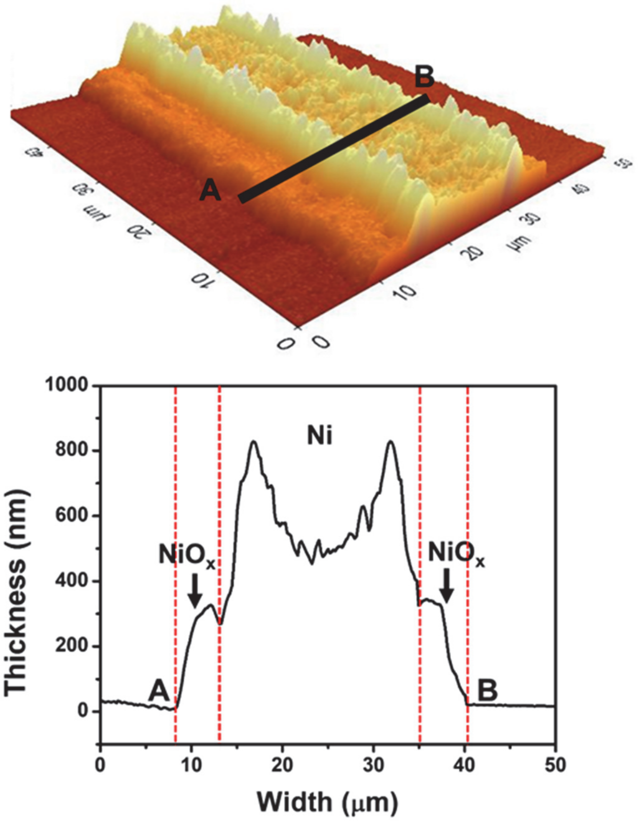

Figure S3. Atomic force microscopy (AFM) image of the single Ni/NiO$_x$ electrode.

Fig. S3 shows the AFM image of a single electrode that was generated from single scanning of the laser beam; the width of that electrode was measured at approximately 5 μm. However, each NiO$_x$ electrode as the sensing channel were generated through two scans of the laser beam (the sensing channel (left): one scan was the last laser scan for the left-side Ni electrode area while another scan was the first scan to create the center electrode area, the same scanning procedure was applied for creating the sensing channel (right) between the center electrode are (last scan) and the right-side electrode area (first scan). Thus, the width of each sensing channel will be twice (~10 μm) as that of single scanning of the laser beam.

4. Surface morphologies of the as-prepared NiO_x thin film and the sensing channel

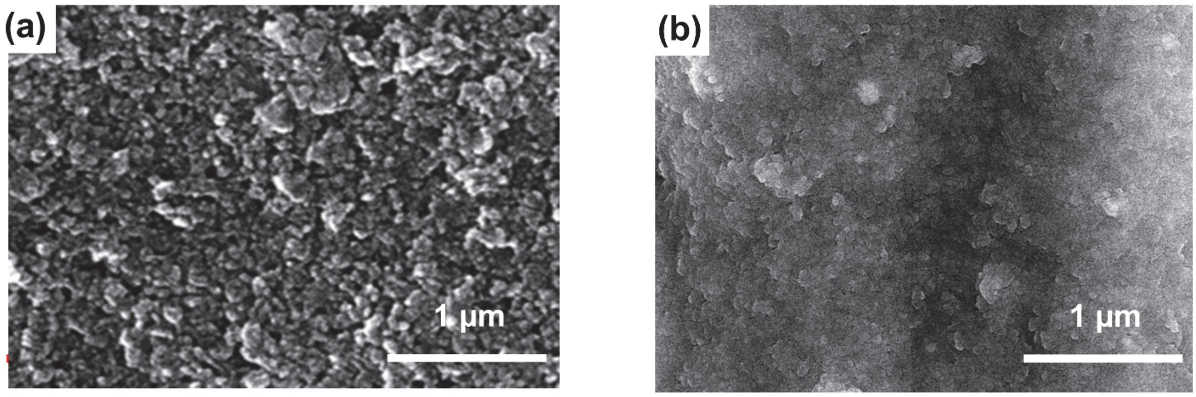

Figure S4. SEM images of (a) the as-synthesized NiO_x thin film and (b) NiO_x as the sensing channel.

5. High magnification SEM images

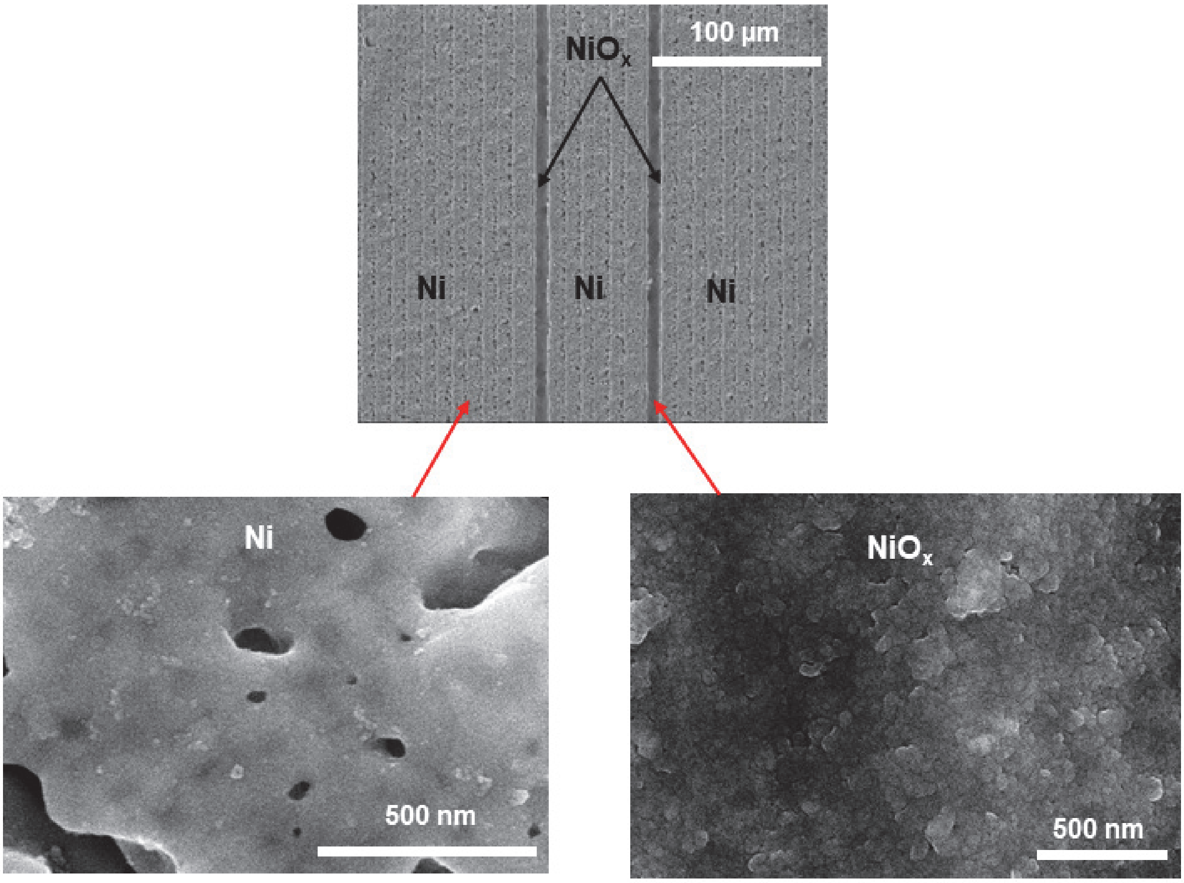

Figure S5. SEM images of the sensor at a high magnification.

6. Schematic illustration of gas sensing mechanism

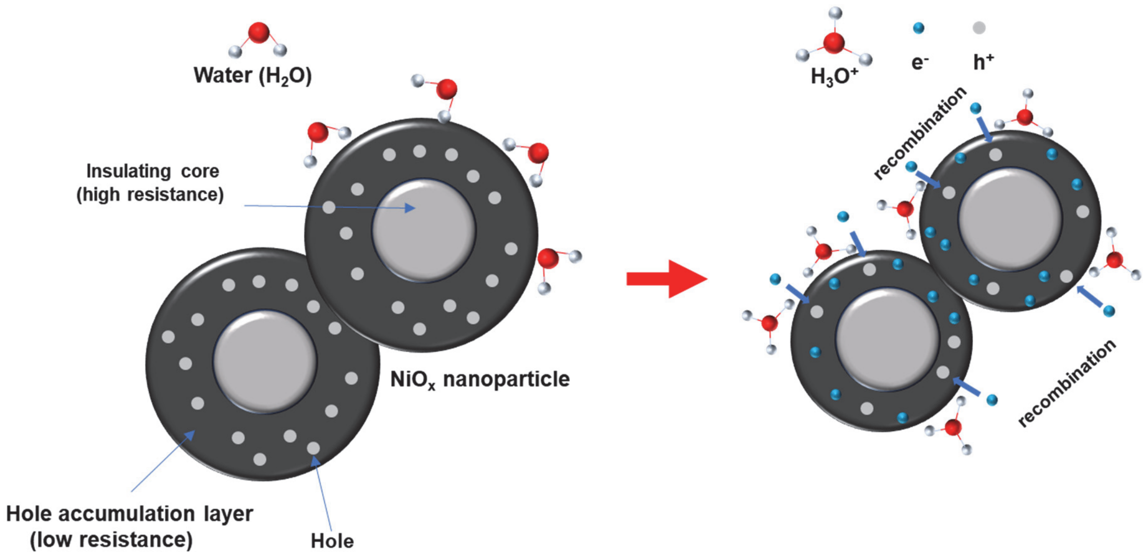

Figure S6. Schematic illustration of humidity sensing mechanism.

7. The performance comparison of the breath sensors for different materials reported in the previous literature and in this study

Table S1. Performance comparison of the breath sensors for different materials reported in the literature and in this study.

Sensing materials	Substrate	Number of layer	Response/recovery times (s)	Ref.
CS-DMV nanofibers	glass	2	2.2/1.05	[1]
ACNT@PU nanofibers	--	2	1.6/1	[2]
VO_2-PDMS	PI	2	0.5/0.5	[3]
MoS_2-coated ESMF	glass	2	0.066/2.395	[4]
rGO	Side-polished fiber (SPF)	1	5/29	[5]
Agarose gel-coated tapered fiber	--	2	5/55	[6]
WS_2	glass	1	1/5	[7]
**NiO_x**	**PI**	**1**	**1.4/1.7**	**This work**

CS-DMV: coronene tetracarboxylate – dodecyl methyl viologen, ACNT@PU: acidified carbon nanotube@polyurethane, PDMS: polydimethylsiloxane, ESMF: etched single-mode fiber, rGO: reduced graphene oxide.

8. The trend of resistance change of the sensor when CO_2 gas and humidity

(1) The effect of CO_2 gas on the sensor performance was tested. The sensor was in a small chamber, and CO_2 gas was injected into the chamber from the CO_2 diffuser. The increased resistance of the sensor is due to the reaction of the NiO_x sensing layer with CO2 molecules to form nickel carbonate and nickel hydroxyl carbonate, thereby increasing the resistance of the sensor [8,9]

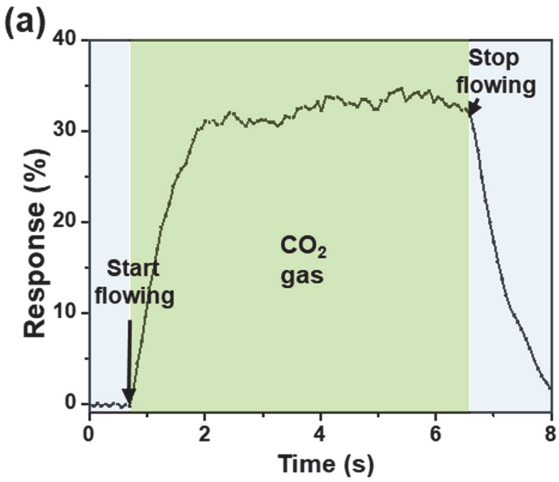

Figure S7. (a) Response of the breath sensor to CO_2 gas

(2) The effect of humidity on the sensor performance was tested. The water vapor flowing from a humidifier was applied on the sensor surface. The response signal of the sensor to humidity was fluctuated because the flow rate from the humidifier was not constant. However, the increase in the response of the sensor to water vapor could be verified.

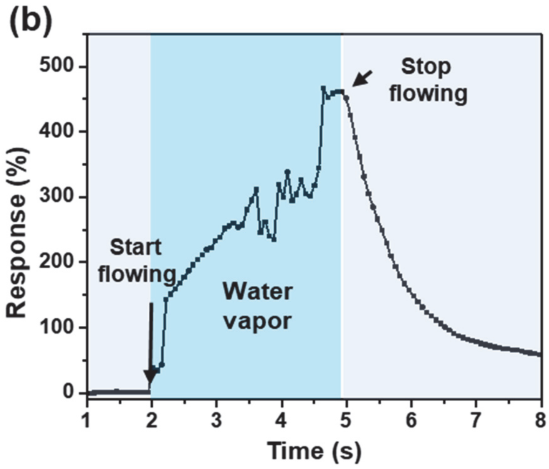

Figure S7. (b) Response of the breath sensor to humidity

Video S1. A video clip showing the response of the sensor to the human breath. (provided separately)

References

1. Mogera, U.; Sagade, A.A.; George, S.J.; Kulkarni, G.U. Ultrafast response humidity sensor using supramolecular nanofibre and its application in monitoring breath humidity and flow. *Sci. Rep.* **2014**, *4*, 1-9.
2. Huang, X.; Li, B.; Wang, L.; Lai, X.; Xue, H.; Gao, J. Superhydrophilic, underwater superoleophobic, and highly stretchable humidity and chemical vapor sensors for human breath detection. *ACS applied materials & interfaces* **2019**, *11*, 24533-24543.
3. Liao, F.; Zhu, Z.; Yan, Z.; Yao, G.; Huang, Z.; Gao, M.; Pan, T.; Zhang, Y.; Li, Q.; Feng, X. Ultrafast response flexible breath sensor based on vanadium dioxide. *J. Breath Res.* **2017**, *11*, 036002.
4. Du, B.; Yang, D.; She, X.; Yuan, Y.; Mao, D.; Jiang, Y.; Lu, F. MoS2-based all-fiber humidity sensor for monitoring human breath with fast response and recovery. *Sens. Actuators B Chem.* **2017**, *251*, 180-184.
5. Xiao, Y.; Zhang, J.; Cai, X.; Tan, S.; Yu, J.; Lu, H.; Luo, Y.; Liao, G.; Li, S.; Tang, J. Reduced graphene oxide for fiber-optic humidity sensing. *Optics express* **2014**, *22*, 31555-31567.
6. Bariain, C.; Matías, I.R.; Arregui, F.J.; Lopez-Amo, M. Optical fiber humidity sensor based on a tapered fiber coated with agarose gel. *Sens. Actuators B Chem.* **2000**, *69*, 127-131.
7. Luo, Y.; Chen, C.; Xia, K.; Peng, S.; Guan, H.; Tang, J.; Lu, H.; Yu, J.; Zhang, J.; Xiao, Y. Tungsten disulfide (WS 2) based all-fiber-optic humidity sensor. *Optics express* **2016**, *24*, 8956-8966.
8. Aslani, A.; Oroojpour, V.; Fallahi, M. Sonochemical synthesis, size controlling and gas sensing properties of NiO nanoparticles. *Appl. Surf. Sci.* **2011**, *257*, 4056-4061.
9. Madou, M.J.; Morrison, S.R. *Chemical sensing with solid state devices*; Elsevier: 2012.
